# Effects of Social Support on the Stress-Health Relationship: Gender Comparison among Military Personnel

**DOI:** 10.3390/ijerph16081317

**Published:** 2019-04-12

**Authors:** Chi-Ming Hsieh, Bi-Kun Tsai

**Affiliations:** 1 International Bachelor Program of Agribusiness, National Chung Hsing University, 145 Xingda Rd., South Dist., Taichung 40227, Taiwan; 2 Graduate Institute of Bio-Industry Management, National Chung Hsing University, 145 Xingda Rd., South Dist., Taichung 40227, Taiwan; pktsai@dragon.nchu.edu.tw

**Keywords:** work-related stress, social support, physical and mental health, gender comparison

## Abstract

The purpose of this study was to investigate how the role of workplace social support and gender affect the relationship between work stress and the physical and mental health of military personnel in Taiwan. The analysis results reveal that military personnel expressed significantly high perceptions of work-related stress. Social support from supervisors and colleagues is a crucial factor in buffering the effect of work-related stress on perceived health, and increasing the physical and mental health among military personnel. This study shows that male personnel who perceived higher stress and gained more social support from supervisors and colleagues than female personnel were less likely to have physical and mental issues than female personnel. Managerial implications and suggestions could serve as references in managing work-related stress, enhancing social support occurring in the military workplace, and reducing job dissatisfaction, which in turn improves the health and well-being of military personnel in Taiwan.

## 1. Introduction

Developments in international and cross-strait situations have provoked the restructuring of Taiwan’s military. In addition to organizational downsizing, the number of troops was sharply reduced from 452 thousand in 1997 to 275 thousand in 2010, and it was further decreased to 196 thousand in 2016 to achieve the goal of “a small, but superior, strong, and smart military force” [1,2]. The number of female personnel in Taiwan’s military is 17,134, which accounts for 8.7 percent of the entire military, but only 744 hold a rank higher than major [3]. In the future, Taiwan’s military will heavily rely on troops of volunteer personnel, through the gradual replacement of compulsory personnel, to comprise the main body of the army. However, Taiwan’s military restructuring raises certain issues, such as the possibility of an increase in the work-related stress of voluntary personnel as a result of downsizing. Previous research has established the influence of work-related stress on military personnel [4,5,6]. A Taiwanese study conducted two decades ago discovered that 56.6% of low-level military officers experienced work-related stress, which was significantly higher than that of the general employed population at that time (25%) [7]. In 2015, sixty-seven percent of the general employed population in Taiwan, with those between 30–40 years of age making up a significant portion of that percentage, reported experiencing work-related stress [8]. As the experiences of work-related stress increase following the downsizing and reorganization that has been occurring since 2015, it is unclear how work-related stress will influence the physical and mental health of individuals in voluntary military service. This could be an important issue in soldiers’ health and for the well-being of future generations, which relevant agencies must consider in order to have the reduced military forces maintain a high standard of performance.

Past studies regarding the effects of social support on the stress-health relationship among military personnel in Taiwan have generally investigated single districts, units, and service types, rather than the military as a whole. This is inadequate in both the breadth and depth of the research on the stress-health relationship of military personnel when compared to that of personnel in other industries [9]. A review of the military literature reveals that it is unclear how the role of social support and gender influences the health or moderates the relationships of stress-health among professional military personnel. To fill this gap, this study focused on both female and male military personnel, sampling from military units all over Taiwan, and developed a modeling architecture from relevant literature, including the following factors: work-related stress, social support, and physical and mental health that are experienced by professional military personnel. Thus, the purpose of this study is to investigate how the role of workplace social support and gender affect the relationship between work stress and the physical and mental health of professional military personnel.

### 1.1. Literature Review

This section presents a review of the literature regarding work-related stress, the buffering model of social support, and the moderating effect of gender among professional military personnel.

#### 1.1.1. Work-Related Stress

Work-related stress refers to the stress that an individual experiences in the workplace or due to the characteristics of his/her job [10] and the physiological, psychological, and behavioral changes that occur in an individual when he/she cannot adapt to the work environment [11]. The responses to work-related stress vary from person to person. Both excessively high and excessively low work-related stress can have adverse effects on an individual’s performance: high pressure can cause work performance to decline, and low pressure does not stimulate an individual to perform. Stress occurs when people negatively interpret situations, and it can lead to health problems [12], for instance, psychosocial stress or physical exhaustion could cause a poorer health status. The majority of the previous research has focused on the effects of high pressure on health and work performance [13]. In addition to impacting physical and mental health, work-related stress can also affect interpersonal relationships and reduce productivity [14].

Different demographic and working characteristics can affect the degree of occupational stress [13]. The hierarchical nature of military organizations, with their emphasis on discipline and obedience, translates into a relatively high stress environment. Sun and Hsu (2001) [15] investigated individuals that were employed in voluntary military service and divided their experiences of stress into three categories: (1) situational stress from military work, which can be caused by being on duty for long periods of time, irregular furloughs, the inequitable distribution of duties, and chronic fatigue; (2) interpersonal stress, which refers to strain resulting from relationships with superiors, colleagues, and subordinates in the military; and, (3) individual/family stress, which includes stress resulting from personal concerns and family situations. Cheng (2005) [16] surveyed officers in mandatory military service in Taiwan’s military and found that the most common pressures involve the perceived unreasonableness in the demands of the organization or management, the validity of extrinsic rewards, and poor relationships with management. In an investigation regarding the effects of work-related stress on personnel in mandatory military service, Tang (2008) [17] discovered that low pressure can significantly increase work satisfaction, and that age and family economic status can lead to significant differences in experiences of work-related stress. We modified the work-related stress scales, including situational and interpersonal stress, based on the above-mentioned studies to measure work-related stress among professional military personnel.

#### 1.1.2. Buffering Model of Social Support

Social support is defined as information that leads people to believe that one is cared for and loved, esteemed, and a member of a network of a mutual obligation [18]. Social support also refers to the general feeling of being adequately supported or cared for by others [19]. According to Cohen and Wills (1985) [20], social support can be regarded as an important factor in dealing with job stress by providing dependable interpersonal relationships that result in social inclusion, reassurance, guidance, and material aid. Social support scales include four kinds of social support in the workplace, including emotional support (psychological, emotiona1, and accepted support), instrumental support (instrumenta1 and material support), advice (advice, guidance, and informationa1 support), and companionship (companion and society) [21,22,23,24]. Schwarzer and Leppin (1991) [25] claimed that internal resources (self-esteem, mastery) and external resources (social support) could help people to deal with stressful lives. It has been shown that social support is a critical factor in buffering the negative physical and mental effects of stress [26]. For instance, Crandall (1979) [27] and Larson, Mannell, and Zuzanek (1986) [28] claimed that companionship and friendship could be seen as the central element of social support; thus, doing enjoyable things, together with companions or friends, could elevate psychological well-being. Social support enhances appraisals and coping to the extent that the particular type of social support matches the demands of the stressor [29,30]. Work stress studies assessing a wide range of occupations have found that social support could reduce people’s strain by decreasing the negative effects of occupational stressors [20]. Social support promotes health by protecting people from the adverse effects of stress across various occupations, such as social workers [31], bank employees [32], and professors [33], indicating that the magnitude of stressor-strain correlations decreases as social support increases [20]. The few studies that are related to professional military personnel have rarely focused the effects of workplace social support on the work stress experience [34,35].

Coleman and Iso-Ahola (1993) [36] proposed the theoretical model of stress-buffering that is based on Ensel and Lin’s (1991) [37] buffering models. The theoretical model claims that social support buffers or moderates the effect of perceived stress on people’s mental health, demonstrating that social support could reduce the negative impact of external stressors on mental health. Iso-Ahola and Park (1996) [38] further supported their research regarding the effect of stress on health/illness and social support’s moderation on the stress-health relationship. Subsequent studies have supported this [39,40,41,42,43,44,45,46]. The buffering model of social support suggests that social support from others could strengthen people’s perceived ability to manage a stressor with effective coping strategies, which will, in turn, improve their health and well-being [33].

Based on the empirical findings of previous research, we formulated the following hypotheses:

**H1:**  The work-related stress experienced by military personnel has a negative impact on their physical and mental health.

**H2:**  The social support obtained by military personnel has a positive impact on their physical and mental health.

**H3:**  The social support obtained by military personnel moderates the relationship between work-related stress and physical and mental health.

#### 1.1.3. Moderating Effect of Gender

The moderating effect of socio-demographics, such as gender in different occupations, has been tested on the relationship between work stress and health. For instance, female teachers with higher stress perceived lower satisfaction with their physical health than male teachers [47]. In another example, female employees in a high-tech industry who perceived more social support from colleagues and supervisors were more satisfied with their work-life balance than the male employees [48]. A few studies in different occupations (e.g., officers, military personnel, junior high school teachers, tour guides) discovered significant differences when adopting moderator variables, such as years of service, in the strength of a causal relationship between the leisure constraints and leisure participation [49,50,51]. Senior officers, for example, were more adept at arranging their leisure time [51]. The extent to which social support and health are perceived by employees undertaking a job within different types of work environments may vary by gender. For instance, males often dominate police and military personnel; primary and secondary school teachers, nurses, and flight attendants are generally female-dominated careers. Over 50 percent of flight attendants that are employed in commercial airlines are females who are more prone to breast cancer and miscarriage than other occupations [52]. Occupational diversity could lead to different results when making gender comparisons. For instance, some studies have indicated that male teachers had higher work-related stress than female teachers, especially intrapersonal stress [13,47,53], with Lin (2009) [54] and Yeh (2009) corroborating these findings [48], who found that female teachers and employees who received higher social support felt healthier than male teachers and employees. Prior studies also conducted the comparative analysis of well-being [55], stress and mental disorders [56], and the effect of stress on job functioning [57] between female and male personnel. Prior studies have analyzed and found that the discriminatory work environment could be a stressor that influences employees’ health [58,59]. Women represent a small proportion of the military in most countries, and they often encounter problems of discrimination and harassment. Based on the above discussion, the gender moderator variable can lead to varying results in work-related stress, social support, and health. Accordingly, we propose the following two hypotheses:

**H4:**  The gender of military personnel moderates the relationship between work-related stress, and physical and mental health.

**H5:**  The gender of military personnel moderates the relationship between social support, and physical and mental health.

**Figure 1:**
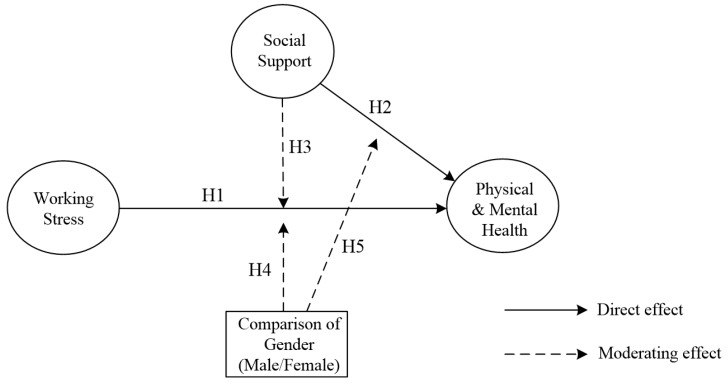
The Proposed Conceptual Model.

This study examined the work-related stress, social support, and physical and mental health that are experienced by military personnel, the relationships between them, and the moderating effects of social support and gender on these pathway relationships. Figure 1 displays the research framework.

## 2. Materials and Methods

Grounded in the research hypotheses that were formulated from the preceding literature review, this study focused on officers, non-commissioned officers, and privates in active voluntary military service in Taiwan’s military. The study adopted the three dimensions that are mentioned above, and hypothesized that the amount of social support that military personnel perceive and/or receive while undergoing the stressful process of acculturation could have an effect on physical and mental health. The measurement instrument included three constructs with 18 corresponding items that were revised from prior studies. The question items focused on the selected variables, with adjustments being made in accordance with the research focus (military service), including: (1) work-related stress, with six question items [15,16,17]; (2) social support, with six question items [22,23,24]; and, (3) physical and mental health, with six question items [38,60,61].

This study adopted a closed-question design with a five-point Likert scale, ranging from 1 (strongly disagree) to 5 (strongly agree). We employed Structural Equation Modelling to test the relationships between three constructs, and hierarchical regression analysis to determine the moderating effects of social support and gender. To increase the content validity, a pilot test was conducted on the first draft, and the wording of question items was modified with the assistance of experts and eight officers in active service. Using snowball sampling, we asked five officers in active service to serve as investigators and recruit other officers. The completed questionnaires were collected and mailed to the researchers by the officer that was in charge of the survey at each military base. We heeded the suggestion by Hair et al. (2006) [62], that the number of valid samples for multivariate analysis should exceed 200. We also considered the suggestion that the number of samples should be twenty times that of the observed variables, as suggested by Hwang (2003) [63]. With 18 question items, we therefore endeavored to recover more than 360 valid questionnaires.

### 2.1. Procedure and Participants

The formal questionnaire survey was administered for a total of two months from March to May in 2017. We distributed a total of 600 questionnaires to investigators’ working units and their friends who worked in other units by the snowball sampling through our five investigators who served in the military. After eliminating 13 invalid questionnaires, this study recovered a total of 398 valid questionnaires, thereby presenting a valid recovery rate of 66.3%. Using SPSS 23 (IBM, Armonk, NY, USA), we performed an item analysis and a reliability analysis. Furthermore, we used a hierarchical regression analysis to test the moderating effect of social support and gender on the relationship between stress and health. During the survey period, we also provided one consent form with the ethical guidelines addressing the requirements (e.g., confidentiality, voluntary participation) for protecting the interests of our research subjects at any time (e.g., data collection, analysis interpretation, publication).

This study found that males (51.8%) were slightly more represented more than females (48.2%) (Table 1). In the largest age group, 42.5% of the respondents were between 21 and 25 years of age, followed by 31.9% ranging between 26 and 30 years of age. The marital status of the vast majority (82.9%) was single. The educational background of the largest group was comprised of senior high school or vocational high school diplomas (36.2%), followed by university graduates (34.9%); 17.1% had graduated from junior colleges, and 11.1% had a master’s degree or higher. The military rank consisted of 33.2% sergeants, 29.4% second lieutenant or above, and 25.9% privates. Many (32.2%) had served between three years and four years, 19.8% had served between one year and two years, 15.8% had served less than a year, and 11.1% had served 11 years or more. The largest group (42.2%) received a personal income between US $1001 and US $1333 per month, followed by 37.4% receiving between US $1334 and US $1667, and 8.8% who received between US $1668 and US $2000 per month.

### 2.2. Instruments and Materials

A summary of the measurement reliability of the three constructs that were formed by 13 indicator variables, using Cronbach’s alpha, is presented in Table 2. The mean scores of six stress indicator variables ranged from 3.71 to 3.25; three indicator variables—“I felt listless when I was on duty”, “I felt incompetent at my current job”, and “I frequently felt exhausted due to my heavy workload”,—reached the highest level of agreement. Three of six social support items, including “My colleagues were friendly and supportive”, “My supervisors frequently provided me practical suggestions to handle problems”, and “My supervisors actively cared for me and understood the difficulties I faced at work”, achieved the highest scores. Among statements related to physical and mental health, “I frequently felt fatigued and/or lacking energy”, “I frequently felt pain”, and “I have had times when I feel particularly low or down for a period of time” had the highest level of agreement. The tests showed that all values of Cronbach’s alpha were greater than 0.7, as recommended by Fornell and Larcker (1981) [64].

## 3. Results

### 3.1. Measurement and Structural Models

The proposed H1–H2 hypotheses were assessed through the measurement and structural models while using Amos 23. The confirmatory factor analysis was first assessed with three constructs. All of the standardized factor loadings ranged from 0.76 to 0.91, which were all higher than the 0.7 threshold, and all of the t-values were significant (*p* < 0.01), supporting the convergent validity [64], as presented in Table 3. The coefficients of composite reliability ranged from 0.86 to 0.91, which were higher than 0.70, indicating acceptable internal consistency reliability [64]. The coefficients of average variance extracted (AVE) ranged from 0.68 to 0.69, which were higher than 0.50, implying that the 18 observed variables have a high accuracy in determining the three latent variables [64]. The discriminant validity was assessed by comparing the square root of the AVE for a given construct with the correlation coefficients between the construct and the other two constructs, as seen in Table 4. The construct validity and reliability are supported after the assessments of both convergence validity and discriminant validity.

The model fit indices for the structural model reached an acceptable standard, with x^2^/df = 2.73, Adjusted Goodness of Fit Index (AGFI) = 0.91, Non-Normed Fit Index (NNFI) = 0.92, Comparative Fit Index (CFI) = 0.91, Standardized Root Mean Residual (SRMR) = 0.07, and Root Mean Square Error of Approximation (RMSEA) = 0.07. Table 5 presents a summary of the model fit indices comprising both the measurement and structural model. Finally, the H1–H2 hypotheses were supported with a positive relationship after assessing the structural model (Table 6). The analysis revealed that working stress negatively affects physical and mental health (β = −0.23, t = 2.01, *p* < 0.05), and social support positively influences working stress (β = 0.38, t = 2.74, *p* < 0.01), thereby supporting H1 and H2, as presented in Table 6.

### 3.2. Modeating Effect of the Gender

**Figure 2:**
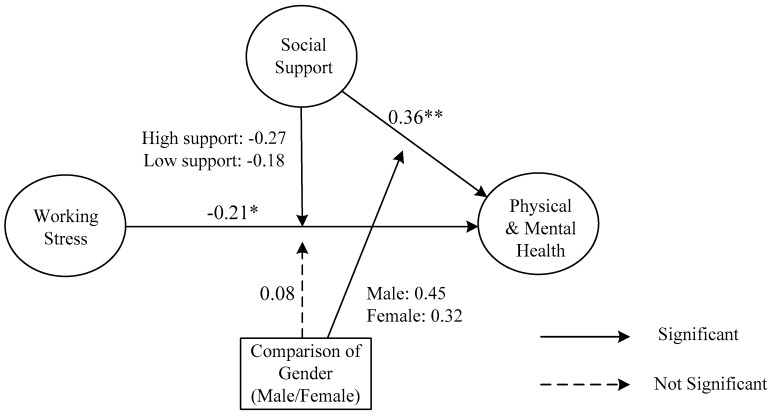
Summary of Tested Hypotheses (H1–H5). (-) refers to the sign of the proposed hypotheses as negative. * *p* < 0.05; ** *p* < 0.01.

Hierarchical regression analyses were then adopted to test the moderating effect of social support on the relationship between working stress, and physical and mental health (H3); the moderating effect of gender on the relationship between working stress and physical and mental health (H4); and, the moderating effect of gender on the relationship between social support and physical and mental health (H5). The main effects of work-related stress and social support were entered in the first step of each moderated hierarchical multiple regression, and the corresponding interaction effects were entered in the second step. The results in Table 7 show that the interaction effect between working stress and social support (working stress × social support) is significant (*β* = −0.29, *p* < 0.05; R^2^ = 0.39, ΔR^2^ = 0.31). The interaction effect between social support and gender social support (social support × gender) is also significant (*β* = 0.43, *p* < 0.01; R^2^ = 0.44, ΔR^2^ = 0.36), indicating that only H3 and H5 are supported. Specifically, the effect of working stress on physical and mental health was stronger in the high social support group (*β* = −0.27, *p* < 0.01) than the effect in the low social support group (*β* = −0.18, *p* < 0.05). In addition, the effect of social support on physical and mental health was stronger in the male group (*β* = 0.41, *p* < 0.01) than the effect in the female group (*β* = 0.32, *p* < 0.05). Figure 2 shows the results of the three moderating effects.

## 4. Conclusions and Suggestions

We aimed to understand the sources and magnitudes of work-related stress and social support among military personnel, and their satisfaction with their physical and mental health. It is our hope that the findings of this study can assist Taiwan’s military in maintaining the combat capabilities of their human resources by improving the quality of life for their personnel, so as to achieve the objectives of the organizational transformation. Managerial implications and potential interpretations that are drawn from these significant findings are provided below; recommendations and suggestions are also offered.

First, military personnel expressed significantly high perceptions of work-related stress with “felt listless on duty”, “felt incompetent at current job”, and “felt exhausted due to heavy workload”. This demonstrates that the primary sources of work-related stress for military personnel were exhaustion or incompetence, which was consistent with earlier studies [65]. Their experiences of work-related stress also increased with rank and the number of years in service. This is consistent with the results that were obtained by Feng (2006) [66] and Wu (2006) [67], who identified the heavy workload as the cause of participants’ stress, although the results were contrary to those of Turnage and Spielberger (1991) [68], who found that the longer the senior teachers worked, the less stress they experienced. This indicates that Taiwan’s military personnel often feel physically and mentally exhausted and anxious due to excessive workloads, which increases their working stress. In addition, military personnel who experience a low sense of work achievement under a high degree of work-related pressure may have interpersonal constraints, such as a lack of companions to accompany them in social or leisure activities. Downsizing has increased the working scopes and the volume of military personnel over the past few years, resulting in more work-related stress than before. Next, the test results showed that the social support buffers the impact of work-related stress on personnel’ physical and mental health. This could be the potential additional effects of the economic crisis in the dynamics of stress-health relationship [69]. Specifically, military service has long been a path for social and economic mobility, and the Armed Forces offers monetary and educational benefits for young people [70]. The Army promotes people based on their performance evaluation, capability, and potential. The working stress in this study primarily includes “I felt unimportant within my department” and “I felt helpless when others criticized my performance at work” may cause the fear of the economic crisis. If the military personnel has a poor performance review, this evaluation may have an influence on the promotion and salary level. Consistent with Giorgi et al. (2015) [71], this study supports that the economic stress in the military workplace may have impact on personnel’ physical and mental health. Drawn from the significant findings, it is recommended that reducing the negative effect of employee downsizing on working performance would be an effective step towards decreasing their work-related stress. For instance, less workplace bullying or harassment in the military could decrease mental health problems among employees, as suggested by previous studies [72]. Military managers should place importance on improving aspects such as establishing proven management techniques of task allocation to prevent the overlapping of tasks or assignments that were carried out by the same units/personnel; and, to form a sound management system featuring clearly specified powers and responsibilities.

Second, this study demonstrates that social support from supervisors and colleagues is a crucial factor in buffering the effect of work-related stress on perceived health, and increasing physical and mental health among military personnel. Consistent with prior studies [39,43,73], the level of social support from supervisors and colleagues that military personnel experienced had a positive and direct influence on the rate of physical and mental health. Consequently, social support is not only identified as the primary driver affecting physical and mental health, but it also plays a moderating role in buffering the negative effects of working stress on military personnel’s physical and psychological well-being. Real support from supervisors and colleagues could eliminate the adverse effects of work-related stress to improve well-being and life quality for military personnel. Hence, military managers should be aware of their personnel’s rights, needs, and feelings, as these can be fundamental factors in increasing that personnel’s satisfaction with their health. It is recommended that military personnel in the same unit could develop and maintain appropriate companionship and solidarity with their supervisors and colleagues to alleviate work-related stress and improve their health satisfaction. Military policy-makers and mangers could promote educational programs, as well as health counseling and training among military personnel, in an effort to create a positive work environment. In addition, the analysis results show that the magnitude of work-related stress that is experienced by the group with high social support was weaker than that experienced by the low social support group and, as a result, the perceived degree of health among the high social support group was stronger than that of the low social support group. Supervising units should enhance their instrumental and informational support based on the individual needs of military personnel, so they can gain more useful advice, guidance, knowledge, and skills while they are on duty. Additionally, unit managers could promote group leisure activities (e.g., outdoor leisure activities) within the same unit after work, to bring cohesion and unity into that unit or improve health, which has been recently implemented by the Ministry of National Defense in Taiwan [74]. The above recommendations could serve as references for improving and managing or reducing work-related stress, enhancing social support occurring in the military workplace, and reducing job dissatisfaction, which, in turn, improves the health and well-being of military personnel.

Finally, we found significant differences when comparing the average scores of female and male military personnel for work-related stress, social support, and perceived health. This study indicated that the average scores for work-related stress and social support of the male group were higher than those of female personnel, but the degree of perceived health of the female group was lower than that of the male group. Consistent with the study of Huang (2007) [47] and Kuo (2004) [48], this study shows that male personnel who perceived higher stress and gained more social support from supervisors and colleagues were less likely to have physical and mental issues than female personnel. The most likely explanation for this result is that differences may exist in the extent to which the stress, social support, and health are perceived by employees undertaking a job within different types of work environments. Traditionally, Taiwan’s military is a male-dominated organization and the males dominate the higher ranks in the military, which may increase the social support from male supervisors among male personnel. Thus, male personnel could seek social support and professional guidance from supervisors and colleagues of the same sex when facing frustration, predicaments, and pressure. The buffering effects of social support may be more likely to reduce male personnel’s stress and increase their physical and psychological health when compared to female personnel. On the other hand, factors such as gender stereotyping, role conflict, and other structural characteristics against female employees [75,76] could lead to a lower perception of social support and health as compared to male personnel. We also conclude that four factors may lead to female personnel’s high stress, low social support, and poor perceived health status in Taiwan’s military. First, females who join the military often carry the primary financial burden for their families, and they endure both working or family pressure stemming from the traditional role of the housewife. Second, since supervisors are mostly males that may not show consideration toward, or may even misunderstand, female subordinates, this leads to weak social support for female personnel in Taiwan’s military. Third, female personnel gain limited social support from female supervisors and colleagues due to the small number of females in the same unit. Last, the counseling currently organized for female personnel is still in a preliminary stage, limiting its contribution to strengthening female’s social support. We suggest that strengthening workplace health and safety, reducing discrimination and harassment, and enhancing fairness, trust and organizational justice through military units, are likely to help female personnel to have sound minds and bodies. Especially, the regulation and procedures for handling sexual harassment has been strictly enforced since 2016 to decrease the cases of sexual harassment or assault [77], and to provide female military personnel a secured workplace [78]. The implementation of this study could provide some insights on the role of gender in male-dominated organizations, such as the military sector in Taiwan and other countries, and some cross-cultural/national research on a well-developed topic based on existing previous literature.

This study is subject to several limitations. The available resources made convenience and snowball sampling the most appropriate choice for this study, and therefore the subjects were limited to certain ranks in Taiwan’s military. Thus, the results may not necessarily be generalizable to all of Taiwan’s military. We suggest that future studies adopt sampling methods of a wider range (such as systematic or random sampling), based on the actual numbers and nature of the population, to reduce sampling errors and enhance the generalizability, comparability, and comprehensiveness of the results. Furthermore, the model in this study is grounded on previous empirical evidence, and the questionnaire survey method was the primary approach. Including qualitative methods for data collection in future studies could increase the depth of the data and the representativeness of the research content. Finally, the analysis results of past studies indicate that construct variables and interactions other than those that were examined in this study may exist. These represent areas that require further investigation.

## Figures and Tables

**Table 1:** Respondents’ profile (*N* = 398).

Characteristics	Categories	Frequency (*N*)	Percentage (%)
Gender	Male	206	51.8
	Female	192	48.2
Age	20 years or less	38	9.5
	21–25 years old	169	42.5
	26–30 years old	127	31.9
	31–35 years old	44	11.1
	36–40 years old	20	5.0
Marital status	Single	330	82.9
	Married	68	17.1
Education	Junior high school or less	3	0.8
	Senior or vocational high school	144	36.2
	Five-Year Junior College	68	17.1
	College	139	34.9
	Graduate school	44	11.1
Military rank	Major (or above)	33	8.3
	Second lieutenant (or above)	117	29.4
	Master sergeant	13	3.3
	Sergeant	132	33.2
	Private	103	25.9
Service years	Less than 6 months	36	9.0
	7–12 months	27	6.8
	1–2 years	79	19.8
	3–4 years	128	32.2
	5–6 years	40	10.1
	7–8 years	25	6.3
	9–10 years	19	4.8
	11 years or above	44	11.1
Personal income	≤US $667	8	2.0
	US $668–1000	17	4.3
	US $1001–1333	168	42.2
	US $1334–1667	149	37.4
	US $1668–2000	35	8.8
	US $2001 or more	21	5.3

**Table 2:** Summary of item content and reliabilities.

Construct and Item	Mean	Std. Deviation	Cronbach’s Alpha
Working Stress			0.88
1. I frequently felt exhausted due to my heavy workload	3.43	0.76	
2. I felt anxious about my current work	3.25	0.88	
3. I felt incompetent at my current job	3.62	0.93	
4. I felt listless when I was on duty	3.71	1.01	
5. I felt unimportant within my department	3.28	0.98	
6. I felt helpless when others criticized my performance at work	3.29	0.87	
Social Support			0.84
7. My supervisors actively cared for me and understood the difficulties I faced at work.	3.55	0.93	
8. My supervisors frequently provided me practical suggestions to handle problems	3.58	0.89	
9. My colleagues were friendly and supportive.	3.73	0.74	
10. My colleagues were caring of each other	3.52	0.87	
11. My department provided adequate support to help me complete tasks.	3.30	0.83	
12. My department offered adequate psychological counseling to help relieve work-related stress.	3.41	0.86	
Physical and Mental Health			0.91
13. I had trouble sleeping and/or didn’t feel well-rested when I woke up.	3.26	0.75	
14. I frequently felt pain (e.g., chest, legs or entire body)	3.41	0.85	
15. I frequently felt fatigued and/or lacking energy	3.53	0.82	
16. I frequently felt nervous and stressful	3.06	0.74	
17. I have had times when I feel particularly low or down for a period of time	3.34	0.77	
18. I felt there was no hope in the future.	2.97	0.76	

**Table 3:** Measurement Model (*N* = 398).

Construct & Indicators	SFL	t-Value	CR	AVE
Working stress			0.93	0.69
1. I frequently felt exhausted due to my heavy workload	0.77	11.62		
2. I felt anxious about my current work	0.81	14.21		
3. I felt incompetent at my current job	0.79	11.34		
4. I felt listless when I was on duty	0.83	10.11		
5. I felt unimportant within my department	0.86	9.38		
6. I felt helpless when others criticized my performance at work	0.91	10.12		
Social Support			0.93	0.68
7. My supervisors actively cared for me and understood the difficulties I faced at work.	0.78	11.25		
8. My supervisors frequently provided me practical suggestions to handle problems	0.76	10.96		
9. My colleagues were friendly and supportive.	0.81	9.15		
10. My colleagues were caring of each other	0.88	13.27		
11. My department provided adequate support to help me complete tasks.	0.83	9.08		
12. My department offered adequate psychological counseling to help relieve work-related stress.	0.87	11.92		
Physical and Mental Health			0.93	0.68
13. I had trouble sleeping and/or didn’t feel well-rested when I woke up.	0.79	12.34		
14. I frequently felt pain (e.g., chest, legs or entire body)	0.84	9.18		
15. I frequently felt fatigued and/or lacking energy	0.77	14.41		
16. I frequently felt nervous and stressful	0.81	13.67		
17. I have had times when I feel particularly low or down for a period of time	0.85	12.29		
18. I felt there was no hope in the future.	0.89	10.54		

Note: SFL = Standardized Factor Loading (λ), CR = Composite Reliability, and AVE = Average Variance Extracted.

**Table 4:** Results of Discriminant Validities and Correlations.

Construct	1. Working Stress	2. Social Support	3. Physical & Mental Health
1. Working Stress	**0.83** ^1^		
2. Social Support	0.25 ***	**0.82**	
3. Physical & Mental Health	0.34 ***	0.38 ***	**0.82**

^1^ The bold values of the diagonal indicate the square root of Average Variance Extracted (AVE); Correlations are the values off the diagonal; *** *p* < 0.001.

**Table 5:** Goodness-of Fit Indices of Measurement and Structural Models.

Model	x²/df	AGFI	NNFI	CFI	SRMR	RMSEA
Measurement model	2.68	0.92	0.93	0.92	0.06	0.06
Structural model	2.73	0.91	0.92	0.91	0.07	0.07
Recommended level	<3.00	≥0.90	≥0.90	≥0.90	<0.08	<0.07

Note: Recommended level: x^2^/df < 3; the Adjusted Goodness of Fit Index (AGFI) ≥ 0.90; the Non-Normed Fit Index (NNFI) ≥ 0.90; the Comparative Fit Index (CFI) ≥ 0.90; the Standardized Root Mean Residual (SRMR) < 0.080; and the Root Mean Square Error of Approximation (RMSEA) < 0.07.

**Table 6:** Results of Tested Hypotheses H1–H2.

Research Hypothesis	Hypothesized Path	Path Coefficient	t-Value	Results
H1	Working stress → Social support	−0.23	−2.01 *	Supported
H2	Social support →Physical & Mental Health	0.38	2.74 **	Supported

* *p* < 0.05; ** *p* < 0.01.

**Table 7:** Summary of Hierarchical Regression Analyses for Moderating effects of Social Support and Gender.

Predict Variable	Step 1	Step 2	Step 1	Step 2	Step 1	Step 2
	Beta (β)	Beta (β)	Beta (β)	Beta (β)	Beta (β)	Beta (β)
Working stress	−0.21 *	−0.57 ***	−0.25 *	−0.42 **		
Social support	0.36 **	0.66 ***			0.23 **	0.38 ***
Gender			0.24 ***	0.16	0.28	0.13
Working stress * Social support		−0.29 *				
Working stress * Gender				0.08		
Social support * Gender						0.43 **
R^2^	0.08	0.39	0.12	0.41	0.43	0.44
ΔR^2^		0.31	0.04	0.33	0.35*	0.36

* *p* < 0.05; ** *p* < 0.01; *** *p* < 0.001; Dependent variable: Physical and mental health.
